# Inflammatory changes in the prepuce and clinical findings according to the stages of phimosis

**DOI:** 10.55730/1300-0144.5963

**Published:** 2024-11-13

**Authors:** Gül DOĞAN, Dilek YILMAZ, Hülya İPEK, Mehmet METİN, Hande KAHRAMAN, Çağatay Evrim AFŞARLAR

**Affiliations:** 1 Department of Pediatric Surgery, Faculty of Medicine, Hitit University, Çorum, Turkiye; 2 Department of Pathology, Sancaktepe Şehit Prof. Dr. İlhan Varank Training and Research Hospital, University of Health Sciences, İstanbul, Turkiye; 3 Department of Medical Microbiology, Faculty of Medicine, Hitit University, Çorum, Turkiye

**Keywords:** Circumcision, children, phimosis, Kikiros grade

## Abstract

**Background/aim:**

Circumcision refers to the surgical removal of all or part of the prepuce. Circumcision is commonly performed on males in childhood to prevent urinary tract infections and for religious reasons. This study was designed to investigate the inflammatory processes in the prepuce according to the degree of phimosis in patients who underwent circumcision.

**Materials and methods:**

We conducted a prospective study on 173 male children under the age of 18 who underwent circumcision between June and September 2024. A comprehensive physical examination of the genitourinary system was performed. Foreskin retraction was assessed and recorded using the Kikiros grading score, and an elective surgical appointment was scheduled accordingly.

**Results:**

As the age of the patient decreases, phimosis appears to be more advanced. In Stage 2 phimosis, where the foreskin is more covered, mild to moderate inflammation is observed, whereas in Stage 1 phimosis, where the foreskin is moderately covered, severe inflammation is noted. This suggests that the partial constriction in Stage 1 phimosis may facilitate contamination from external sources.

**Discussion:**

It is known that circumcision in boys reduces urinary tract infections. This study investigated the inflammatory processes in the prepuce according to the degrees of phimosis. The results indicate that performing circumcision at younger ages is beneficial for preventing ascending urinary infections and reducing the need for antibiotic treatment.

## 1. Introduction

The circumcision procedure involves surgical removal of part or all of the prepuce (foreskin) of males [1]. Although the exact origins of circumcision, one of the oldest surgical techniques practiced, are not fully known, hieroglyphs depicting circumcision rituals have been found on ancient Egyptian walls dating back to around 4000 BC [2]. Circumcision is one of the most widely practiced surgical interventions worldwide. While approximately 60% of males born in the United States undergo circumcision, nearly every male in Türkiye is circumcised [3]. Approximately 30% of the total male population worldwide has undergone circumcision [4]. In regions with a significant population where circumcision is not traditionally practiced, such as in China, India, and Japan, the proportion reaches 33% when circumcisions performed for health or sociocultural reasons are included [5]. Medical indications for circumcision include phimosis, paraphimosis, recurrent urinary tract infections (UTIs), and the need for clean intermittent catheterization [6]. While in the Western world circumcision is often performed to prevent sexually transmitted diseases or penile cancer, in Türkiye and other Muslim-majority countries, it is performed due to religious beliefs and traditions. In recent years, there has been a rapid increase in circumcision rates in non-Muslim communities for reasons other than these indications [5,6]. Recent studies suggest that a significant majority of UTIs are related to the presence of the foreskin, and UTIs are claimed to be 10–20 times more common in uncircumcised boys[7,8]. This is because the bacterial colonization that occurs under the foreskin can be transmitted to the urinary system. Circumcision helps to prevent this colonization, thereby reducing the risk of UTIs [9]. This study was designed to investigate the inflammatory processes in the prepuce in male patients undergoing circumcision according to the degree of phimosis.

## 2. Materials and methods

A prospective study was conducted on male pediatric patients under the age of 18 who underwent circumcision between June and September 2024. Patients who required concomitant surgical procedures were not included in this study. During the initial admission, patient demographics were noted, caregivers were asked about UTI history, urine culture results were documented, and patient histories of balanitis or balanoposthitis were taken. A detailed physical examination of the genitourinary system was performed, foreskin retraction was graded and recorded according to the Kikiros score (Table 1), and an elective surgical appointment was given [10].

### 2.1. Surgical procedure

All of the circumcisions were performed under general anesthesia by four attending pediatric surgeons. Before cleaning of the surgical area, the foreskin was retracted to obtain a swab culture from the glans penis adjacent to the external urethral meatus, which was sent to the microbiology laboratory for culturing. Then, the surgical area was cleaned with a 10% povidone–iodine solution and covered with a sterile surgical drape. A pudendal nerve block was done with a bupivacaine hydrochloride (0.25%, 0.3 mL/kg) injection. A standard surgical circumcision was performed, and the foreskin specimen was sent for histopathology examination.

### 2.2. Histopathology study

The foreskin specimens sent for routine histopathology examination were fixed in 10% neutral formaldehyde for 12 h and embedded in paraffin. Four-micrometer-thick sections were obtained, mounted on slides, and stained with hematoxylin-eosin. The case specimens showed common patterns in terms of inflammation status, inflammation severity, and distribution of inflammatory cells (Figure). Inflammation score groups and histopathological features created on this basis are as follows:

Score 1: No inflammation;
Score 2: At the level of the papillary dermis, a small number of rare lymphocyte clumps fill the dermal papilla (minimal inflammation);
Score 3: There is lymphocytic infiltrate in the superficial dermis around the vascular structures or surrounding the sebaceous glands at the mid-dermis level (mild-to-moderate inflammation);
Score 4: There is lymphocyte-dominant lichenoid inflammatory infiltrate beneath the epithelium (severe inflammation).

### 2.3. Statistical methods

All statistical analyses were performed using SPSS 22.0 software (IBM Corporation, Armonk, NY, USA). The normality of the data distribution was assessed using the Kolmogorov–Smirnov and Shapiro–Wilk tests. Descriptive statistics for normally distributed data were presented as means and standard deviations, while categorical variables were summarized as frequencies and percentages. The homogeneity of variances across groups was evaluated using Levene’s test. Comparisons among three independent groups, assuming normality and homogeneity of variances, were conducted using one-way analysis of variance (ANOVA). Relationships between categorical variables were examined using Fisher’s exact test. Statistical significance was defined as p < 0.05 for all analyses.

## 3.Results

During the study period, 173 patients were eligible for the study. The mean age of the patients was 5.15 ± 2.6 years. In order to make a practical analysis, the Kikiros foreskin retraction grading was modified so that the patients were grouped into phimosis grade 0 (Kikiros grades 0 and 1), phimosis grade 1 (Kikiros grades 2 and 3), and phimosis grade 2 (Kikiros grades 4 and 5).

There was a statistically significant difference in terms of age distribution and phimosis grade in that as age increases, phimosis grade decreases (p < 0.001) (Table 2).

There was a significant difference between inflammation score and patient age (p < 0.001) (Table 3). The mean age of patients with an inflammation score of 3 was significantly lower that of patients with inflammation scores of 1, 2, and 4 (p = 0.003, p = 0.003, p = 0.043, respectively). There was no significant difference among the inflammation scores of 1,2, and 4 (p > 0.05).

There was a significant difference between inflammation score and phimosis grade (p < 0.001) (Table 4). Inflammation scores of 3 and 4 were more common, and inflammation scores of 1 and 2 were less common, for phimosis grade 1 as compared to phimosis grade 0. Comparing phimosis grade 2 to phimosis grade 0, the inflammation scores 2 and 4 did not vary significantly, but inflammation score 3 was considerably higher and inflammation score 1 was significantly lower in phimosis grade 2 (Figure).

The swab cultures were reported as distal urethral flora bacteria or no growth in 96% of the patients, and as *Escherichia coli* or *Enterococcus faecalis* in 4% of the patients. There was no significant difference between the balanitis and swab culture results among the phimosis grades (p = 1.000, Fisher’s exact test).

## 4. Discussion

Circumcision remains one of the most commonly performed surgeries worldwide due to both medical and religious/traditional reasons [3]. In some countries it is traditionally performed in the first week after birth, while in others it is carried out at any time regardless of age. However, in Türkiye, it is typically performed before adolescence. According to a study conducted by Kestel et al., the ages of patients applying for circumcision in Türkiye ranged from 5 to 7 years old. Consistent with the literature, the mean age of patients in this study was 5.15 ± 2.6 years [11]. Studies have shown that circumcision reduces the risk of UTIs, balanitis, phimosis, and paraphimosis development [8]. It can also be recommended as a therapeutic method for balanitis xerotica obliterans, which can lead to pathological phimosis [9]. Circumcision performed in childhood has been noted to improve penile hygiene and prevent penile inflammation, thereby reducing the risk of invasive penile cancer [12,13]. A statement released by the American Academy of Pediatrics in 2012 stated that circumcision helps prevent the transmission of human immunodeficiency virus (HIV) and certain sexually transmitted diseases, and does not negatively affect penile sensitivity or sexual function [6]. Additionally, the Centers for Disease Control and Prevention has issued a temporary guideline stating that the benefits of circumcision outweigh the surgical risks [7].

It is known that microorganisms residing on the mucosal surface of the foreskin spread to the urinary system primarily through ascending rather than hematogenous routes [14]. In a study conducted by Sonmez et al., *Salmonella typhimurium* was found to colonize the foreskin. In the current study, no pathogenic microorganisms were isolated in 96% of patients, while *Escherichia coli* and *Enterococcus faecalis* were isolated in 4% of patients [15].

A study conducted by Kayaba et al. reported that incomplete retraction of the foreskin is responsible for bacterial colonization that can lead to balanoposthitis and/or UTIs [16]. This current study also reports mild-to-moderate inflammation in foreskins with grade 2 phimosis, where the foreskin is more closed, and severe inflammation with grade 1 phimosis, where the foreskin is moderately closed. This suggests that the partial constriction of grade 1 phimosis facilitates contamination from the external environment.

Since the keratinization of the inner epithelium of the glans and prepuce is not complete at birth, adhesions may occur after neonatal circumcision; however, the frequency of this occurrence decreases with age [17]. In a study by Eroğlu et al., mucosal adhesions and meatal stenosis were detected in circumcisions performed within the first 3 months [18]. Altay et al. noted that the frequency of phimosis decreases with increasing age [19]. Consistent with the literature, this study found that the grade of phimosis decreased with increasing age. Patients with an inflammation score of 3 were found to be significantly younger than the others. Since the prepuce is typically closed in newborns, the risk of external contamination and inflammation is lower. Therefore, unless there is an urgent need, it may be more appropriate not to rush circumcision in this age group. For other age groups, this research suggests that circumcision can be beneficial in preventing ascending urinary tract infections and reducing the need for antibiotic treatment.

## Figures and Tables

**Figure:**
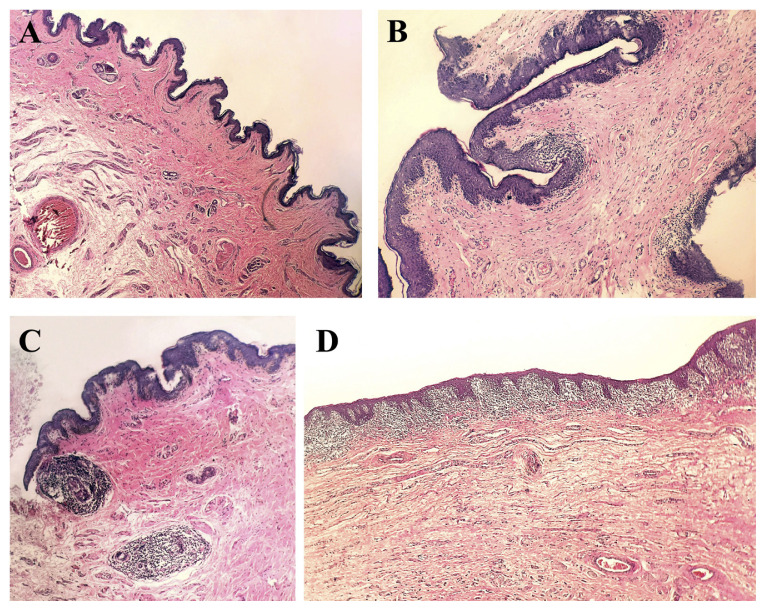
**(A)** Preputium tissue covered with stratified squamous epithelium without any inflammation including rich vascular structures and peripheral nerve sections (**Score 1**) (HE ×40). **(B)** Sparsely distributed lymphoid cell clusters occasionally filling the papillary dermis in the subepithelial area (**Score 2**) (HE ×200). **(C)** Inflammation observed around the skin appendages at the surface and around vascular structures in the mid-dermis (**Score 3**) (HE ×100). **(D)** Preputium with acanthosis in the epithelium and band-like lichenoid inflammation under the epithelium (**Score 4**) (HE ×200).

**Table 1:** Grades of foreskin retractability according to Kikiros et al. [10].

Grade	Definition
**0**	Full retraction, not tight behind glans, or easy retraction limited only by congenital adhesions to the glans
**1**	Full retraction of foreskin, tight behind the glans
**2**	Partial exposure of glans, prepuce (not congenital adhesions) limiting factor
**3**	Partial retraction, meatus just visible
**4**	Slight retraction, but some distance between tip and glans, i.e., neither meatus nor glans can be exposed
**5**	Absolutely no retraction

**Table 2:** Comparison of phimosis grades and patient age distribution.

Phimosis grade	N	Age (year) Mean ± SD	Minimum	Maximum	p value	Posthoc p values
0	115	5.70 ± 2.51	0.01	12.40	**<0.001** £	**0–1: 0.008** **0–2: 0.003**
1	39	4.28 ± 2.40	0.03	8.92		
2	19	3.64 ± 2.56	0.13	8.30		
Total	173	5.15 ± 2.60	0.01	12.40		

£ = ANOVA was statistically significant;

SD = standard deviation.

**Table 3:** Comparison of inflammation scores and patient ages.

Inflammation score	N	Age (year) Mean ± SD	Min	Max	p value	Posthoc p values
**1**	32	5.92±2.57	1.64	11.81	**<0.001** £	**1–3: 0.003** **2–3: 0.003** **3–4: 0.043**
**2**	75	5.53±2.77	0.13	12.40		
**3**	54	3.96±2.18	0.01	7.36		
**4**	12	6.12±1.28	4.88	8.41		

£ = ANOVA was statistically significant;

SD = standard deviation.

**Table 4:** Comparison of inflammation scores and phimosis grades.

			Inflammation score				Total	p value
			1	2	3	4		
**Phimosis grade**	**0**	n	30	53	25	7	115	**<0.001** *
		%	26.1	46.1	21.7	6.1	100.0	
	**1**	n	1	13	21	4	39	
		%	2.6	33.3	53.8	10.3	100.0	
	**2**	n	1	9	8	1	19	
		%	5.3	47.4	42.1	5.3	100.0	
**Total**		n	32	75	54	12	173	
		%	18.5	43.4	31.2	6.9	100.0	

* = Fisher’s exact test was statistically significant.
